# Multiple Factors Involved in the Pathogenesis of White Matter Lesions

**DOI:** 10.1155/2017/9372050

**Published:** 2017-02-21

**Authors:** Jing Lin, Dilong Wang, Linfang Lan, Yuhua Fan

**Affiliations:** ^1^Department of Neurology, Guangdong Key Laboratory for Diagnosis and Treatment of Major Neurological Diseases, National Key Clinical Department, National Key Discipline, First Affiliated Hospital of Sun Yat-sen University, Guangzhou 510080, China; ^2^Department of Medicine and Therapeutics, Prince of Wales Hospital, The Chinese University of Hong Kong, Shatin, Hong Kong

## Abstract

White matter lesions (WMLs), also known as leukoaraiosis (LA) or white matter hyperintensities (WMHs), are characterized mainly by hyperintensities on T2-weighted or fluid-attenuated inversion recovery (FLAIR) images. With the aging of the population and the development of imaging technology, the morbidity and diagnostic rates of WMLs are increasing annually. WMLs are not a benign process. They clinically manifest as cognitive decline and the subsequent development of dementia. Although WMLs are important, their pathogenesis is still unclear. This review elaborates on the advances in the understanding of the pathogenesis of WMLs, focusing on anatomy, cerebral blood flow autoregulation, venous collagenosis, blood brain barrier disruption, and genetic factors. In particular, the attribution of WMLs to chronic ischemia secondary to venous collagenosis and cerebral blood flow autoregulation disruption seems reasonable. With the development of gene technology, the effect of genetic factors on the pathogenesis of WMLs is gaining gradual attention.

## 1. Introduction

The term cerebral small vessel disease (CSVD), which encompasses all the pathological processes of the cerebral small vessels, has frequently referred to cognitive impairment and dementia. Most often, CSVD is used to only arterial vessels. Venules and capillaries, however, also belong to CSVD and should get more attention. The consequences of CSVD on the brain parenchyma mainly show lacunar infarcts, cerebral microbleeds (CMBs), white matter lesions (WMLs), and enlarged perivascular spaces (EPVS). Previous studies have found that multiple factors are associated with CSVD, but the most common risk factors are hypertension and age [1]. Recent researches also suggested that salt intake plays a key role in the formation of WMLs through the effect on endothelial cells [2, 3]. Despite improvements in radiological markers available for the characterization of CSVD, none can view the pathological changes of small vessels directly and there remain other factors yet to be identified.

**Figure 1:**
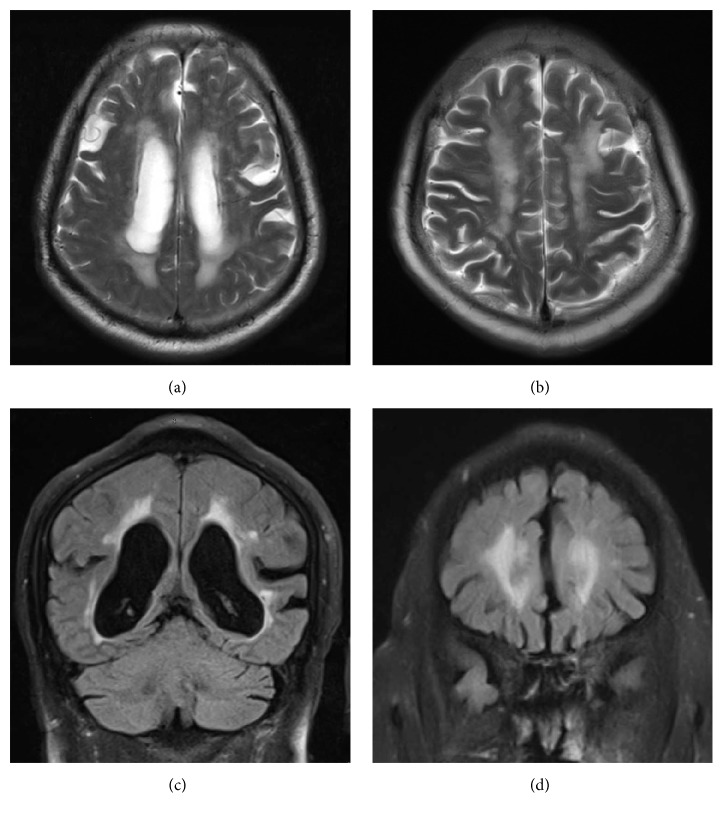
Neuroimaging features of white matter lesions: (a) periventricular white matter lesions on T2-weighted MRI. (b) Deep/subcortical white matter lesions on T2-weighted MRI. (c) Periventricular white matter lesions on MRI (FLAIR image). (d) Deep/subcortical white matter lesions on MRI (FLAIR image). FLAIR: fluid-attenuated inversion recovery.

WMLs, as a subtype of CVSD, are also called leukoaraiosis (LA) or white matter hyperintensities (WMHs). Their main manifestations are hyperintensities on T2-weighted or FLAIR images (Figure 1). With the aging of the population and the development of imaging technology, the incidence of WMLs in elderly individuals has gradually increased. The pathological features of WMLs are mainly pale myelin, demyelination, oligodendrocyte apoptosis, and vacuolization [4]. WMLs clinically manifest as cognitive decline, dementia, abnormal gait, and urinary incontinence [5–8]. Although WMLs are gradually attracting more attention, their pathogenesis is still unclear. This paper reviews the following five aspects of the pathogenesis of WMLs: classification and anatomical characteristics, cerebral blood flow autoregulation, venous collagenosis, blood brain barrier disruption, and genetic factors.

## 2. Classification and Anatomical Characteristics of WMLs

### 2.1. Classification of WMLs

According to MRI features, WMLs are classified as periventricular WMLs and deep/subcortical WMLs (Figure 1). Periventricular WMLs include the smooth caps around the frontal horns and the line-like and halo-like lesions along the bodies of the lateral ventricles. Deep/subcortical WMLs, however, include punctuate or confluent lesions that are distant from the lateral ventricle. Different types of WMLs may have varying pathogeneses.

### 2.2. Blood Supply Watershed due to Anatomy

Periventricular white matter receives its blood supply mainly through the long perforating branches and ventriculofugal vessels. The ventriculofugal vessels are the terminal branches of the choroidal or striatal arteries of the subependymal arteries, and they sparsely coincide with the long perforating branches of the cerebral pial vasculature to form a blood supply watershed; these anatomical characteristics make the periventricular white matter susceptible to ischemic damage [9, 10]. Comparatively, the subcortical white matter receives its blood supply primarily through the short branch arteries originating vertically from the long perforating branches when near the subcortical white matter. The long perforating branches have a long and often tortuous course. Most short branches originate only from a long perforating branch. Taken together, these anatomical characteristics make the subcortical white matter also susceptible to hypoxic-ischemic damage [9]. The proximal cortical U-shaped fiber, however, is supplied with blood by the long perforating branch nutrient arteries supplying white matter and the short branch arteries supplying cortex, where ischemic WMLs rarely occur [9].

## 3. Cerebral Blood Flow Autoregulation and the Pathogenesis of WMLs

White matter is located in the watershed area of the arterial blood supply that is susceptible to ischemic damage. Furthermore, in a PET study, ischemia in white matter regions was revealed by an increase in the proportion of oxygen uptake in those regions [11]. In animal research, cerebral hypoperfusion in 2-vessel occlusion (2-VO) animal model can be induced by occlusion of bilateral common carotid arteries, while white matter is located in the watershed area, which is susceptible to ischemic injury [12]. The findings of the studies mentioned above all suggest that WMLs originate from ischemia; however, the causes of ischemia are still unclear. Recent studies have found that hemodynamic changes may be involved in the ischemia of white matter [13–16]. Impaired cerebral blood flow autoregulation is the most common type of hemodynamic change.

### 3.1. Diffuse Impairment of Dynamic Cerebral Autoregulation in Cerebral Small Vessel Disease

Dynamic cerebral autoregulation (dCA) has varying characteristics among the different subtypes of acute ischemic stroke. The dCA processes of large artery atherosclerosis and small artery occlusion, the two most common subtypes of acute ischemic stroke, differ significantly. Studies of large artery atherosclerotic stroke found that ipsilateral dCA damage was more severe than contralateral impairment [17–20], whereas, for small artery occlusion stroke, the damage to ipsilateral and contralateral dCA was equally severe [18]. This finding may be due to the hypoperfusion caused by aortic stenosis on the ipsilateral side of a large artery atherosclerotic stroke, which results in angiectasis, such that the ability of vessels to expand is relatively poor. In contrast, small artery occlusive dCA reflects extensive cerebral small vessel disease such that a decline in dCA is present on both the ipsilateral and the contralateral sides of the stroke. By assessing cerebral blood flow autoregulation, in which the bilateral middle cerebral arterial and posterior cerebral arterial dCA processes represent the dCA of the entire brain, Guo et al. found that dCA damage in cerebral small vessel disease was not limited merely to unilateral or bilateral effects but involved the entire brain [21]. Small cerebral vascular sclerosis, stiffness and luminal stenosis caused by long-term hypertension, diabetes mellitus, or other vascular risk factors may be the primary causes of the impairment of cerebral vascular autoregulation throughout the brain.

### 3.2. Reciprocal Connection of WMLs and AD through Cerebral Blood Flow Autoregulation

WMLs are the MRI marker for cerebral small vessel disease. The severity of WMLs is closely associated with Alzheimer's disease (AD), and the reciprocal connection between cerebral small vessel disease and AD is often mediated by cerebral blood flow autoregulation [22, 23]. Current studies generally support the concept that AD is due to progressive neuronal death, which is primarily attributed to the deposition of A*β* toxic substances [24]. Impaired cerebral blood flow autoregulation that is caused by cerebral small vessel disease, either directly or indirectly, promotes the deposition of A*β* and influences the clearance of A*β* [25–27]. Conversely, A*β* can also influence cerebral blood flow autoregulation by damaging endothelial cell function and promoting vascular wall stiffness [28, 29].

## 4. Venous Collagenosis and the Pathogenesis of WMLs

Previous investigations have focused on the changes of cerebral arteries rather than veins. WMLs are often accompanied by pathological changes in small arteries, such as wall thickening and luminal stenosis [30, 31]. Hyaline wall thickening of the long penetrating arterioles and impaired autoregulation may result in ischemia damage of the white matter. Since Moody et al. proposed the concept of periventricular venule collagenosis in 1995 [32], the effects of venous collagen remodeling and the venous system on WMLs have begun to be taken seriously.

### 4.1. Venous Ischemia

Previous studies found that carotid artery stenosis is closely related to WMLs [33, 34]. Chuang et al. found that incomplete circle of Willis may contribute to WMLs in patients with carotid artery stenosis, and restoration of cerebral perfusion by carotid artery revascularization can reduce WMLs severity [35]. Some studies, however, have shown that the degree of carotid stenosis is not related to the severity of WMLs [36, 37]. Patankar et al. found no association between WMLs and resting cerebral blood flow in patients with severe occlusive/stenotic disease of the extracranial arteries and attributed WMLs to impairment of autoregulation [38]. Compared with arterial ischemia, venous ischemia should gain more attention. In venous ischemia, vasogenic edema and blood brain barrier (BBB) damage are more prevalent. Moreover, venous ischemia is a long-term and more indolent process. In addition, the pathological features and progression caused by venous ischemia are more similar to those of WMLs [39]. Changes in the cerebral parenchyma caused by carotid stenosis or occlusion are often attributed to unilateral vascular lesions. In contrast to arterial diseases, the obstruction of unilateral jugular vein output often causes limited venous drainage in the bilateral deep vein system, superficial venous system, and watershed area due to venous reflux to the superior sagittal sinus or the transverse sinus. These conditions result in bilateral WMLs and clinical findings that better resemble those associated with bilateral WMLs [40, 41]. Therefore, venous ischemia should be involved in the pathogenesis of WMLs and needs to get more attention.

### 4.2. Periventricular Venule Collagenosis

Recent studies also support the concept that periventricular venule collagenosis is associated with WMLs [32, 42]. In an autopsy study of 22 patients aged 60 years or older that used alkaline phosphatase (AP) staining for arteriovenous differentiation, Moody et al. found that 13 patients had periventricular venule collagenosis; of these patients, 10 patients with severe periventricular venule collagenosis had statistically significant WMLs [32]. Although the reason for the association between venous collagen disease and WMLs is unclear, Moody et al. attributed it mainly to genetic predisposition.

### 4.3. JVR and PWE

“Jugular venous reflux (JVR)” and “pulse wave encephalopathy (PWE)” have been recently proposed as being involved in WMLs [43, 44]. JVR refers to spontaneous jugular venous reflux during the Valsalva maneuver or rest, and its primary cause is the pressure difference between the bilateral jugular venous valves and incompleteness of valves. Pathophysiological changes resulting from JVR-induced intracranial venous hypertension may be a cause of WMLs. Pathophysiological changes may include BBB damage, hypoperfusion, venule collagenosis, cerebral blood flow autoregulation impairment, and endothelial cell dysfunction [43, 45–47]. The concept of PWE proposes that, with aging, vascular dysfunction manifests not only as ischemia but also as hemodynamic abnormalities driven by pulse wave changes [44]. In addition to cerebral vascular stenosis or occlusion, age-related changes of pulse waves also play an important role in the damage of microcirculation especially in cerebral venules [48–51].

## 5. BBB and the Pathogenesis of WMLs

The central nervous system, especially neurons, needs a stable environment. Maintenance of neuronal stability depends mainly on the BBB, which consists of fenestration-free endothelial cells with tight junctions, basement membrane, and perivascular endfeet of astrocytes [52]. Regarding the pathological features of cerebral small vessel disease, Fisher observed the anatomical structures of the deep perforating arteries and noted the presence of diffuse abnormalities in cerebral small blood vessels, calling them “segmental arteriolar disorganization.” This disorganization does not simply refer to cerebral small vascular wall thickening or luminal stenosis but to the loss of the normal outer membrane and smooth muscle layer structure and to BBB damage and other abnormalities [31, 53].

### 5.1. Incomplete Lacunar Infarcts in Cerebral Small Vessel Disease

Lacunar infarcts in CSVD are present along lenticulostriate arteries rather than only at the arterial terminal. Pathologically, these lacunar infarcts are often incomplete infarcts. In addition, edema is often present around the deep perforating arteries [54, 55]. These findings suggest that lacunar infarcts in cerebral small vessel disease may not be complete infarcts caused by arterial occlusion but, rather, are incomplete infarcts caused by BBB damage.

### 5.2. BBB Permeability in WMLs

Similarly, WMLs, another subtype of cerebral small vessel disease, may also be associated with BBB damage. The entry of secondary serum substances, such as serum proteins, complement components, and fibrinogens, into the cerebral parenchyma after BBB damage may also underlie the pathogenesis of WMLs. Starr et al. used contrast-enhanced MRI and found that, compared with normal subjects, contrast agents leaked more in the perforating arterial areas of patients with WMLs [56]. Similarly, Wallin et al. used the CSF/serum albumin ratio to represent BBB permeability and found that white matter hyperintensities were associated with BBB permeability [57]. Young et al. reduced the number of cases and the bias from selecting histopathologically different regions, and their MRI findings showed the presence of BBB damage in both WML and non-WML regions, thus further illustrating the close association between WMLs and BBB damage [58]. In animal studies, the association between WMLs and BBB damage has also been demonstrated. A study in the stroke-prone spontaneously hypertensive rat (SHRSP) found that BBB damage to white matter occurred before subcortical ischemic changes [59]. In a study in the stroke-prone renovascular hypertensive rat (RHRSP) our research team also found that, with an increasing duration of hypertension, the expression of ZO-1 and occludin (two components of the BBB) decreased gradually, and WMLs became more severe [60]. According to a meta-analysis by Farrall and Wardlaw, BBB permeability was closely associated with the severity of WMLs (5 comparisons, C : S = 122 : 88, SMD 0.60, 99% CI 0.30, 0.89, *p* < 0.01), which further confirmed the correlation between BBB damage and WMLs [61].

### 5.3. BBB Disruption and WMLs: Controversies

Whether BBB disruption is involved in the pathogenesis of WMLs remains controversial. With the development of imaging techniques, particularly the introduction of contrast-enhanced MRI, a series of studies have shown that BBB damage is the cause of WMLs. Topakian et al. detected BBB permeability by contrast-enhanced MRI and found that BBB damage was present not only in white matter hyperintensities but also in seemingly normal white matter, indicating that BBB damage is associated with WMLs, as the cause rather than the effect of WMLs [62]. Huisa et al. revealed that, in Binswanger's disease (more severe WMLs), BBB damage was present not only in the sites of WMLs upon MRI but also in the margins of WMLs and in normal sites, suggesting that BBB damage is closely associated with the progression of WMLs [63].

The association between the BBB and WMLs has been proven by both human and animal studies, and with the development of imaging technology, BBB damage has been confirmed as the cause of WMLs. However, there is also a study that claimed that BBB damage was not correlated with WMLs [64]. Additional unbiased, rational research is needed to confirm the association between WMLs and the BBB.

## 6. Genes and the Pathogenesis of WMLs

With the development of gene technology, studies of WMLs have begun to focus on genetic factors. Although it is generally considered that the most important risk factors for WMLs are aging and hypertension [65, 66], genetic factors also play an important role in WMLs, possibly in as many as 55%–80% of cases [67, 68].

### 6.1. Genetic Linkage Studies and Genetic Susceptibility Studies

Genome-wide linkage analyses on WMLs in the past few decades have found that WMLs were linked to chromosome 4 [69], chromosome 5 [70], chromosome 1 [70, 71], and chromosome 11 [72]. Meanwhile, studies on the genetic susceptibility of WMLs have fallen into two categories: candidate gene association studies (CGAS) and genome-wide association studies (GWAS). CGAS have identified a large number of WML-related genes that are involved in a series of biological processes, such as ApoE regulating cholesterol [73]; ACE, AGT, and AGTR1 regulating blood pressure and cerebral blood flow [74, 75]; the regulation of immune reactions and inflammatory mediators (IL-6 and IL5RA) [76, 77]; BDNF regulating neuron regeneration [78]; MMP family members regulating neuroinflammatory [79, 80]; and PON1/NOS3 regulating oxidative stress [77]. GWAS have not only identified WML-related susceptibility genes but also initiated research on the molecular mechanism of WMLs. The Cohorts for Heart and Aging Research in Genomic Epidemiology (CHARGE) Consortium reported a GWAS meta-analysis on WMLs, which confirmed that a locus in long arm region 25 of chromosome 17 involving seven known genes had six WML-related new SNPs; these seven genes mainly involved the neural immune and inflammatory systems. Among the six new SNPs, TRIM65 presented the strongest association with WMLs, while TRIM65 was involved in the pathophysiological process of apoptosis [77]. Based on the gene studies on WMLs, the Medical Research Council Cognitive Function and Aging Study (MRC-CFAS) pointed out that the pathogenesis of WMLs might involve activation of multiple cellular pathways and molecular processes including oxidative stress and inflammation. This provided a new way to study WMLs.

Studies concerning CGAS, GWAS, and gene expression have suggested that neural immunity, inflammation, oxidative stress, and apoptosis may be involved in the formation of WMLs. Our research found that long-term treatment with pioglitazone has a beneficial effect on hypertension-induced WMLs, partly through its effect on the attenuation of brain inflammation, which indirectly confirmed the correlation between inflammation and WMLs [81]. A series of cascades induced by chronic ischemia activate a large number of neural inflammatory cytokines, thereby triggering downstream cascades to cause a series of pathophysiological changes, ultimately leading to demyelination, which may be one of the pathogenic mechanisms of WMLs.

### 6.2. Genetic Factors of WMLs in Animal Studies

Animal studies on WMLs have also suggested the involvement of genetic factors in the pathogenesis of WMLs. Lin et al. showed that, with no significant difference in blood pressure, the SHRSP was more prone to WMLs than the spontaneously hypertensive rat (SHR) [82], suggesting the effects of genetic factors. Ogata et al. found that when the SHRSP was used as an animal model for WMLs, WMLs occurred 24 weeks later and became increasingly severe with aging [83]. However, Brittain et al. found that 10-month-old SHRSP had no WMLs [84]. We used the RHRSP as a model for WMLs, in which renal hypertension was induced by 2-kidney, 2-clip methods. When there was no significant difference in blood pressure, not every rat was found to develop WMLs after 20 weeks; moreover, rats with WMLs also exhibited lesions of differing severities (unpublished data). These findings indicate that genetic factors are one of the pathogenic mechanisms of WMLs.

### 6.3. Debates and Prospects of Genetic Factors in WMLs

There are only a few large studies of genes expression on WMLs. A small number of WML genetic linkage analysis studies have suggested that genes are closely associated with WMLs, but the precise gene loci have never been identified. It should be noted that no linkages between genes obtained from genome-wide linkage analyses and WMLs are repeatable; in addition, the associations suggested in CGAS are not identical to the results in GWAS. Therefore, more collaborative longitudinal studies are needed to further investigate the association between genes and WMLs.

## 7. Conclusion

**Figure 2:**
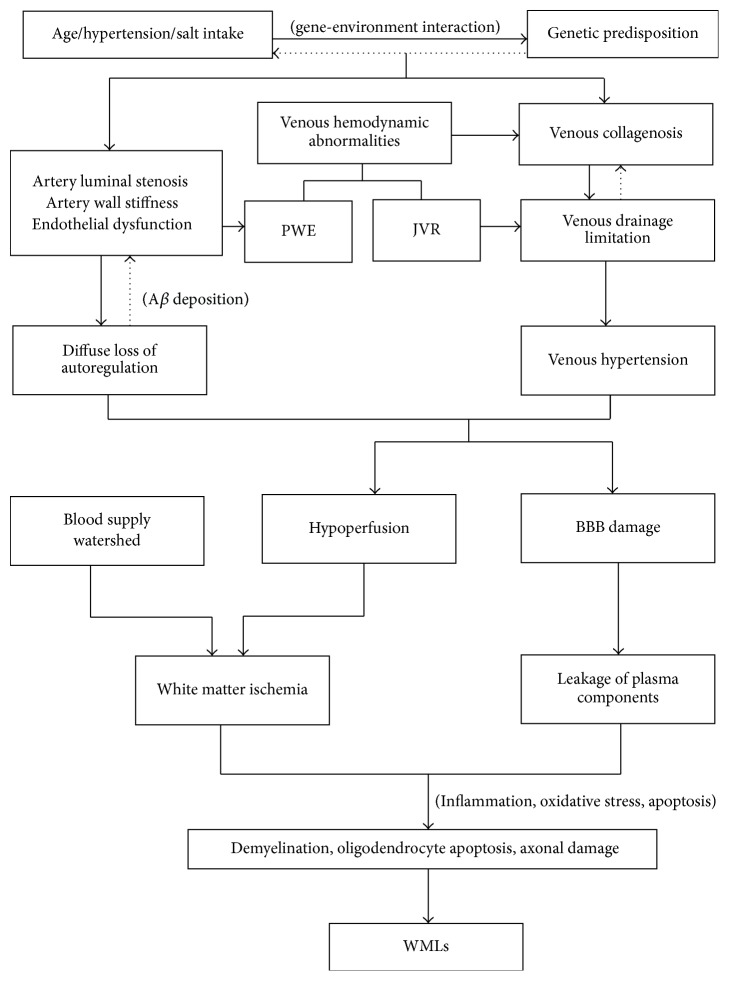
Hypothesis about the pathogenesis of WMLs. JVR: jugular venous reflux; PWE: pulse wave encephalopathy; BBB: blood brain barrier; WMLs: white matter lesions.

Pathogenesis may vary for different types of WMLs. The pathological features of periventricular WMLs support their origin in ependymal layer damage, while the anatomical features of the periventricular white matter arterial blood supply support their origin from ischemia. Thus, periventricular WMLs may be attributable to both ischemia and the ependymal layer, while most studies on subcortical WMLs support long-term chronic ischemia as the cause. Hence, more research is needed to determine whether different types of WMLs have varying pathogeneses. Numerous studies have suggested that WMLs originate from the chronic ischemia. Compared with the simple luminal stenosis, attribution of chronic ischemia to venous collagen deposition and impaired cerebral blood flow autoregulation seems more reasonable. The pathological features of BBB damage suggest its involvement in WMLs. However, the association between BBB damage and WMLs remains controversial. More studies are needed to confirm the correlation between the BBB and WMLs and to explore the mechanisms underlying BBB damage. Research on genes and WMLs has suggested that genes may be involved in the pathogenesis of WMLs; moreover, the characteristics of gene loci further suggest the involvement of neural inflammation and immunity in the formation of WMLs. Multiple factors may be involved in WMLs (Figure 2). More research is needed to confirm the correlations of these factors with WMLs to provide new target treatments for WMLs.
